# Evaluate the photosynthesis and chlorophyll fluorescence of *Epimedium brevicornu* Maxim

**DOI:** 10.1038/s41598-022-24165-x

**Published:** 2022-11-14

**Authors:** Jian Zaiyou, Tang Xiaomin, Wang Hongsheng, Xu Guifang

**Affiliations:** 1 Henan Institute of Science and Technology, Xinxiang, 453003 China; 2 Guangdong Pharmaceutical University, Guangzhou, 510006 China

**Keywords:** Ecology, Physiology

## Abstract

The diurnal variation of photosynthesis, light response curve and CO_2_ response curve in *Epimedium brevicornu* Maxim leaves were determined with Li-6400 photosynthesis system to evaluate the photosynthesis of *E. brevicornu*. Fluorescence of chlorophyll in the leaves were determined with PAM-2500 portable chlorophyll fluorescence apparatus in the study. The results showed that the midday depression of photosynthesis was very obvious in the *E*. *brevicornu* leaves. The light compensation point of *E*. *brevicornu* leaves was about 15 µmol m^−2^ s^−1^. The light saturation point of *E. brevicornu* leaves was below 800 µmol m^−2^ s^−1^, which was lower than the general sunlight intensity at noon in summer. The CO_2_ saturation point of *E. brevicornu* leaves was much higher than the content of CO_2_ in general air. *E. brevicornu* was a typical shade plant and could survive in very low sunlight. *E. brevicornu* could not endure strong sunlight and high air temperature. The net photosynthetic rate of *E. brevicornu* leaves linearly correlated with the content of CO_2_ in the leaf chamber when the content was below CO_2_ saturation point. *E. brevicornu* possessed great potential of photosynthesis.

## Introduction

As a kind of traditional Chinese medicine with aphrodisiac, anti-rheumatic and tonic effects, epimedii folium was usually used to cure impotence, emission, osteomalacia, rheumatism, apoplexy and so on^1^. Epimedii folium was dried leaves preparation from Ep*imedium brevicornu* Maxim, *E*. *pubescens* Maxim, *E*. *sagittatum* (Sicb. et Zucc.) Maxim or *E*. *koreanum* Nakai^1^. There were some medicinal chemical components such as Icariin, Caohuoside, Baohuoside, Epimedin A, Epimedin B and Epimedin C in Epimedii folium^2,3^.

Epimedii folium came from wild resources in the past times. The wild Epimedii folium resources were sharply decreasing because of the increased demand on them and the change of environment. The plants in *Epimedium* are herbaceous perennial^4^. The root and the rhizome of these plants can grow for several years although their leaves wither in winter. The roots and rhizomes of these plants were usually dug out by people because there are certain content of medicinal chemical components in them. Therefore, Epimedii folium resources were seriously destroyed. To satisfy the needs of patient and protect wild Epimedii folium resources, the plants of Epimedii folium should be bred and cultivated.

The diurnal variation of photosynthesis, light response curve, CO_2_ response curve and chlorophyll fluorescence characteristics of *E. brevicornu* leaves were determined in this study to define the suitable growing conditions and provide evidence in support of cultivating *E. brevicornu*. The results contribute to studying the cultivation of *E. brevicornu*, satisfying the needs of patients on Epimedii folium and protecting the wild *E. brevicornu* resources.

## Results

**Figure 1:**
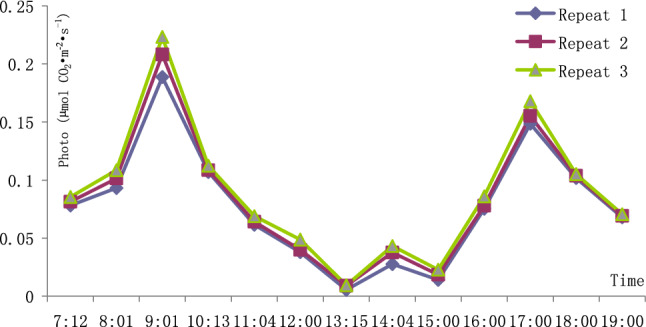
Diurnal variations in *Epimedium brevicornu* leaves photosynthesis.

The results of the diurnal variation of photosynthesis in *E. brevicornu* leaves are shown in Table 1. The diurnal variation curve of photosynthesis in the leaves is drawn in Fig. 1.

**Table 1:** Diurnal variations in *Epimedium brevicornu* leaves photosynthesis.

Repeat	Time	Photo	PARi	Tleaf	CO_2_S	Trmmol	Cond	Ci
1	7:12	0.0780	108.5	28.68	433.0	0.0433	0.000578	372.1
	8:01	0.0931	176.8	31.67	433.0	0.0435	0.000153	371.0
	9:01	0.1888	628.7	33.24	433.1	0.0433	0.000578	201.7
	10:13	0.1070	1460.9	33.53	434.1	0.1344	0.001138	221.6
	11:04	0.0615	1578.3	35.16	427.0	0.1593	0.000865	218.2
	12:00	0.0377	1667.6	36.44	425.4	0.2048	0.001061	262.4
	13:15	0.0053	1598.9	37.25	421.2	0.2590	0.001659	323.1
	14:04	0.0276	1554.4	37.27	416.9	0.1860	0.001404	308.5
	15:00	0.0140	1446.8	36.39	415.3	0.0977	0.000652	295.7
	16:00	0.0752	1163.1	35.08	413.2	0.0487	0.000439	94.0
	17:00	0.1485	934.4	34.01	411.8	0.0586	0.000708	45.7
	18:00	0.1017	544.7	33.16	412.4	0.0353	0.000545	88.2
	19:00	0.0678	58.3	32.28	415.0	0.0367	0.000734	241.0
2	7:12	0.0813	100.6	28.64	433.1	0.0415	0.000558	386.0
	8:01	0.1014	180.7	31.65	433.0	0.0492	0.000223	373.3
	9:01	0.2082	589.5	33.26	433.0	0.0469	0.000596	171.4
	10:13	0.1086	1432.3	33.47	435.1	0.1358	0.000909	171.4
	11:04	0.0642	1578.4	35.17	427.0	0.1590	0.000864	213.7
	12:00	0.0400	1668.4	36.45	425.5	0.2057	0.001063	259.1
	13:15	0.0089	1555.1	37.23	421.0	0.2527	0.002035	338.5
	14:04	0.0376	1554.3	37.27	416.9	0.1857	0.001434	300.6
	15:00	0.0185	1349.0	36.51	414.5	0.0965	0.000773	304.4
	16:00	0.0779	1153.2	35.08	413.1	0.0512	0.000456	94.5
	17:00	0.1552	933.5	34.01	411.8	0.0599	0.000735	44.2
	18:00	0.1037	543.9	33.15	412.4	0.0356	0.000549	84.3
	19:00	0.0691	58.4	32.27	415.0	0.0363	0.000728	236.8
3	7:12	0.0855	105.5	28.56	433.3	0.0363	0.000532	386.1
	8:01	0.1086	178.8	31.65	433.0	0.0363	0.000202	371.2
	9:01	0.2234	591.9	33.26	433.0	0.0437	0.000556	220.4
	10:13	0.1122	1460.8	33.53	434.0	0.1333	0.001142	216.0
	11:04	0.0689	1634.3	35.59	425.6	0.1294	0.000783	201.7
	12:00	0.0488	1667.9	36.44	425.4	0.2057	0.001064	247.9
	13:15	0.0093	1598.9	37.25	421.2	0.2595	0.00165	319.0
	14:04	0.0432	1530.9	37.07	417.4	0.2176	0.001347	276.4
	15:00	0.0228	1397.9	36.32	414.8	0.0999	0.000889	308.7
	16:00	0.0859	1147.0	35.09	413.2	0.0517	0.000458	69.4
	17:00	0.1679	934.3	34.01	411.8	0.0584	0.000711	6.2
	18:00	0.1051	571.0	33.71	412.5	0.0390	0.000498	46.1
	19:00	0.0706	58.4	32.26	415.0	0.0368	0.000738	236.0

Photo, photosynthetic rate, unit: µmol CO_2_ m^−2^ s^−1^. PARi, in-chamber quantum sensor, unit: µmol m^−2^ s^−1^. Tleaf, temperature of leaf thermocouple, unit: °C. CO_2_S, sample cell CO_2_, unit: µmol mol^−1^. Trmmol, transpiration rate, unit: mmol H_2_O m^−2^ s^−1^. Cond, conductance to H_2_O, unit: mol H_2_O m^−2^ s^−1^. Ci, intercellular CO_2_ concentration, unit: μmol CO_2_ mol^−1^.

There is obvious midday depression in the diurnal variation of photosynthesis of *E*. *brevicornu* leaves*.* They commonly photosynthesized in the morning and evening on sunny days in summer. The photosynthesis nearly stopped at noon when the air temperature was high and the sunlight was intense. *E. brevicornu* leaves did not endure the strong sunlight and high temperature.

**Figure 2:**
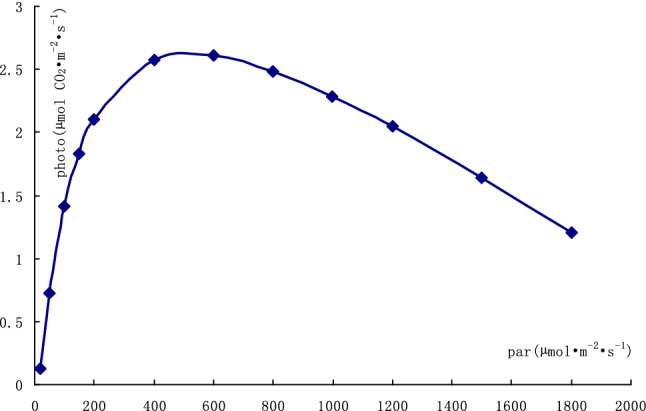
Fitted light response curve of *Epimedium pubescens* leaf photosynthesis with average indexes.

The results of light response curves of photosynthesis in the leaves are shown in Tables 2 and 3. The fitted light response curve of photosynthesis in the leaves with the average fitted indexes in each repeat of light response curve determination is shown in Fig. 2.

**Table 2:** Light response curves of *Epimedium brevicornu* leaves photosynthesis.

Repeat	PARi	Photo	Fitted	CO_2_S	Trmmol	Tleaf	Cond	Ci
1	2000.5	2.005	1.897	400.3	0.670	28.07	0.0496	326.6
	1800.1	2.108	2.050	399.6	0.623	28.09	0.0450	315.6
	1498.9	2.147	2.272	400.2	0.640	27.93	0.0311	277.4
	1399.9	2.177	2.342	400.1	0.539	28.10	0.0380	299.3
	1001.2	2.684	2.600	399.5	0.491	28.12	0.0340	264.1
	801.5	2.705	2.702	400.2	0.454	27.96	0.0369	245.4
	600.8	2.736	2.765	399.8	0.442	28.14	0.0301	245.2
	399.6	2.699	2.738	400.3	0.416	28.14	0.0280	242.6
	199.7	2.570	2.419	399.8	0.396	28.14	0.0264	235.2
	150.8	2.250	2.205	399.3	0.405	28.14	0.0268	238.1
	100.6	1.825	1.833	400.1	0.387	28.15	0.0254	276.1
	49.6	0.925	1.092	399.9	0.400	28.15	0.0261	333.7
	20.5	0.341	0.266	400.7	0.373	28.15	0.0242	368.4
2	2000.1	2.171	2.077	400.1	0.809	28.09	0.0543	326.2
	1800.1	2.374	2.350	400.0	0.719	28.11	0.0477	297.1
	1499.9	2.720	2.713	399.6	0.678	28.12	0.0448	274.9
	1199.1	2.777	2.991	400.0	0.626	28.13	0.0411	284.3
	999.3	3.008	3.102	399.7	0.560	28.14	0.0364	257.9
	799.8	3.213	3.120	400.5	0.531	28.15	0.0345	280.7
	601.0	3.073	2.995	400.4	0.486	28.16	0.0314	255.4
	399.7	2.688	2.617	399.5	0.462	28.16	0.0299	267.7
	199.7	1.904	1.753	399.8	0.414	28.15	0.0269	277.0
	149.8	1.241	1.404	400.8	0.230	27.97	0.0072	330.0
	100.7	0.837	0.978	400.3	0.246	28.05	0.0117	334.3
	49.8	0.338	0.425	399.9	0.256	27.97	0.0123	285.6
	20.5	0.156	0.040	399.4	0.281	28.06	0.0133	335.2
3	1999.9	0.952	0.898	484.7	0.944	28.79	0.0113	297.0
	1800.6	1.226	1.201	483.3	0.964	28.20	0.0135	293.4
	1599.8	1.423	1.497	480.8	0.955	28.35	0.0158	298.1
	1400.8	1.751	1.779	477.7	0.925	27.84	0.0179	288.3
	1200.6	2.004	2.046	474.3	0.892	28.51	0.0202	287.0
	1001.2	2.230	2.286	471.3	0.822	28.29	0.0217	282.7
	800.5	2.570	2.485	468.6	0.751	28.13	0.0233	285.1
	600.7	2.676	2.607	465.8	0.678	28.01	0.0249	288.0
	399.7	2.493	2.571	463.7	0.713	28.24	0.0301	305.6
	200.2	2.164	2.109	463.1	0.779	27.87	0.0330	341.7
	149.6	1.823	1.825	462.3	0.793	27.55	0.0317	353.1
	99.5	1.420	1.398	461.4	0.751	28.16	0.0299	367.9
	49.7	0.617	0.725	461.2	0.702	28.26	0.0286	409.2
	20.8	0.195	0.136	458.8	0.523	28.11	0.0194	423.4

Photo, photosynthetic rate, unit: µmol CO_2_ m^−2^ s^−1^. PARi, in-chamber quantum sensor, unit: µmol m^−2^ s^−1^. Tleaf, temperature of leaf thermocouple, unit: °C. CO_2_S, sample cell CO_2_, unit: µmol mol^−1^. Trmmol, transpiration rate, unit: mmol H_2_O m^−2^ s^−1^. Cond, conductance to H_2_O, unit: mol H_2_O m^−2^ s^−1^. Ci, intercellular CO_2_ concentration, unit: μmol CO_2_ mol^−1^.

**Table 3:** Results of fitting light response curves and CO_2_ response curves.

Project	Repeat	E	M	N	LCP (CCP)	LSP (CSP)	E·LCP (E·CCP)	PLSP (PCSP)	R^2^
Light response	1	0.0536	0.000230	0.01465	14.017	534.52	0.751	2.771	0.979
	2	0.01496	0.000304	0.002298	17.67	863.54	0.264	3.127	0.987
	3	0.0284	0.000389	0.00647	15.314	525.84	0.435	2.620	0.993
	Average	0.03232	0.000308	0.007806	15.667	641.30	0.483	2.839	0.986
CO_2_ response	1	0.01113	0.000309	0.000001	35.56	1634.6	0.396	8.794	0.973
	2	0.01011	0.0002707	0.000001	51.16	1871.0	0.517	9.063	0.982
	3	0.01049	0.000283	0.000001	58.72	1794.6	0.616	8.945	0.988
	Average	0.010577	0.000288	0.000001	48.48	1766.7	0.5097	8.934	0.981

LCP is light compensation point, unit: µmol m^−2^ s^−1^. CCP CO_2_ compensation point, unit: µmol mol^−1^. CSP is CO_2_ saturation point, unit: µmol mol^−1^. LSP is light saturation point, unit: µmol m^−2^ s^−1^. PLSP is the net photosynthetic rate at the light saturation point, unit: µmol m^−2^ s^−1^. PCSP is the net photosynthetic rate at the CO_2_ saturation point, unit: µmol m^−2^ s^−1^.

*E. brevicornu* leaves were able to survive in very low sunlight such as 15 µmol m^−2^ s^−1^. The photosynthesis in the leaves quickly increased along with the increase of light intensity when the light intensity was above the light compensation point. The light saturation point was much lower than the intensity of direct sunlight in summer. Strong sunlight inhibited the photosynthesis in *E. brevicornu* leaves.

The results of CO_2_ response curve of photosynthesis in the leaves are shown in Tables 3 and 4.

**Table 4:** CO_2_ response curves of *Epimedium pubescens* leaves photosynthesis.

Repeat	CO2S	Photo	Fitted	Trmmol	Tleaf	PARi	Cond	Ci
	2563.5	5.3565	5.834	1.043	28.09	1000.9	0.0488	2104.6
	1899.2	9.3139	8.553	0.894	28.13	1001.3	0.0411	1473.1
	1695.6	9.6047	8.781	0.923	28.14	1001.3	0.0426	1277.7
	1398.7	7.8518	8.603	0.971	28.24	1001.2	0.0445	1068.9
	1099.0	7.5999	7.808	1.012	28.07	1001.0	0.0474	825.9
	999.8	7.2034	7.409	1.012	28.18	1000.8	0.0468	720.1
	800.2	6.1402	6.401	1.038	28.24	1000.8	0.0478	567.8
	600.3	5.3804	5.117	1.020	28.06	1001.0	0.0479	400.5
	400.5	3.4728	3.558	0.966	28.17	1000.6	0.0447	262.8
	311.7	2.3457	2.777	0.908	28.13	1000.5	0.0420	212.2
	139.4	1.5264	1.106	0.325	29.06	1199.8	0.0112	80.5
	91.2	0.7625	0.602	0.856	29.55	1200.6	0.0185	20.9
2	2567.3	7.3494	7.740	0.741	28.10	1000.4	0.0345	1987.4
	1897.3	9.4502	9.061	0.667	28.11	1000.1	0.0309	1295.6
	1696.8	9.7506	8.980	0.682	28.11	1000.3	0.0316	1149.0
	1398.4	8.3402	8.453	0.690	28.09	1000.2	0.0321	937.5
	1099.3	7.4384	7.435	0.733	28.10	1000.3	0.0341	715.3
	1000.5	6.8615	6.991	0.712	28.09	1000.2	0.0332	637.9
	800.4	5.6185	5.929	0.720	28.09	1000.3	0.0336	507.4
	601.4	4.2436	4.654	0.697	28.09	1000.2	0.0325	373.7
	399.8	2.7830	3.142	0.641	28.08	1000.1	0.0298	238.1
	310.1	2.2032	2.397	1.014	28.22	1000.6	0.0468	224.3
	113.4	1.4361	0.610	0.322	28.00	1200.3	0.0111	95.2
	88.8	0.3868	0.371	0.920	27.89	1200.9	0.0561	75.4
3	2564.2	6.6075	7.192	0.696	28.08	1000.3	0.0342	2016.5
	1898.3	8.6724	8.913	0.609	28.09	1000.7	0.0477	1352.5
	1696.5	9.6606	8.917	0.585	28.09	1000.1	0.0325	1077.5
	1399.2	8.1282	8.482	0.698	28.10	1000.3	0.0324	952.8
	1099.7	7.3780	7.513	0.705	28.14	999.9	0.0303	805.4
	999.7	6.6075	7.071	0.676	28.04	1000.2	0.0388	685.7
	800.3	6.0660	6.012	1.040	28.17	1001.1	0.0483	572.3
	600.4	4.7405	4.714	1.028	28.18	1000.7	0.0476	421.0
	413.5	3.2650	3.285	1.003	28.14	1000.9	0.0466	287.5
	311.0	2.1327	2.413	0.974	28.14	1001.0	0.0452	225.0
	202.1	1.0168	1.418	0.866	28.13	1199.8	0.0340	146.6
	105.2	0.6105	0.473	0.750	28.03	1200.5	0.0199	51.2
	83.9	0.0484	0.323	0.992	28.75	1200.8	0.0324	77.5

Photo, photosynthetic rate, unit: µmol CO_2_ m^−2^ s^−1^. PARi, in-chamber quantum sensor, unit: µmol m^−2^ s^−1^. Tleaf, temperature of leaf thermocouple, unit: °C. CO_2_S, sample cell CO_2_, unit: µmol mol^−1^. Trmmol, transpiration rate, unit: mmol H_2_O m^−2^ s^−1^. Cond, conductance to H_2_O, unit: mol H_2_O m^−2^ s^−1^. Ci, intercellular CO_2_ concentration, unit: μmol CO_2_ mol^−1^.

**Figure 3:**
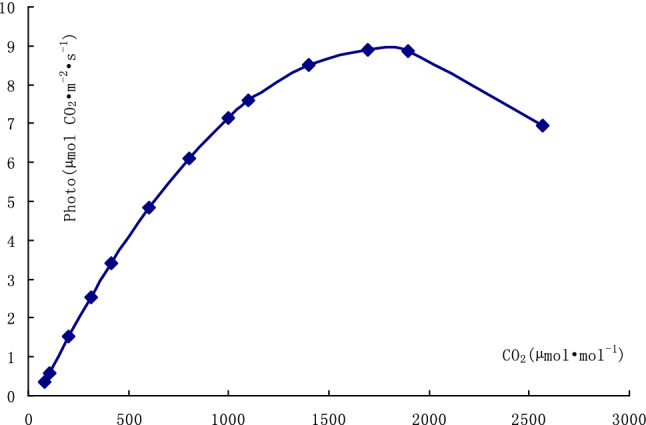
Fitted CO_2_ response curve of *Epimedium pubescens* leaf photosynthesis with average indexes.

The fitted CO_2_ response curve of photosynthesis with the average fitted indexes in each repeat of CO_2_ response curve determination is shown in Fig. 3.

The CO_2_ compensation point was about 48 µmol mol^−1^, which was much lower than the content of CO_2_ in air in field. The photosynthesis in *E. brevicornu* leaves was approximately linear to the content of CO_2_ in the leaf chamber. The CO_2_ saturation point the leaves was about 1766 µmol mol^−1^, which was much higher than the content of CO_2_ in air in field. *E. brevicornu* leaves possessed large potential of utilizing CO_2_ in photosynthesis. The CO_2_ with excessive content inhibited the photosynthesis in *E. brevicornu* leaves.

The results of slow kinetics of chlorophyll fluorescence in the leaves are shown in Table 5.

**Table 5:** Slow kinetics and rapid light curves of chlorophyll fluorescence in *Epimedium pubescens* leaves.

Repeat	Slow kinetics									
	PAR	Y(II)	Y(NPQ)	Y(NO)	qN	qP	ETR	(Fm − Fo)/Fm		
1	198	0.124	0.62	0.256	0.879	0.5	10.3	0.5986		
2	198	0.129	0.595	0.276	0.873	0.57	10.7	0.5630		
3	198	0.137	0.565	0.298	0.851	0.568	11.4	0.5583		
Average	198	0.13	0.593	0.2767	0.868	0.546	10.8	0.5733		

Repeat	Rapid light curves									
	PAR	Y(II)	Y(NPQ)	Y(NO)	NPQ	qN	qP	qL	ETR	Fitted
1	0	0.573	0	0.427	0	0	1	1	0	0
	6	0.36	0.264	0.376	0.701	0.559	0.837	0.745	0.9	0.503
	31	0.184	0.529	0.287	1.846	0.831	0.667	0.592	2.4	2.273
	101	0.124	0.585	0.292	2.005	0.843	0.459	0.383	5.3	5.502
	198	0.098	0.604	0.298	2.027	0.849	0.374	0.306	8.1	7.988
	363	0.066	0.626	0.308	2.034	0.856	0.265	0.213	10.1	10.244
	619	0.049	0.637	0.314	2.027	0.849	0.187	0.145	12.7	12.079
	981	0.032	0.648	0.32	2.027	0.853	0.125	0.096	13.1	13.603
	1386	0.02	0.657	0.323	2.034	0.856	0.078	0.06	14.4	14.839
	2015	0.015	0.664	0.321	2.064	0.856	0.059	0.045	16.6	16.497
	2970	0.015	0.665	0.32	2.08	0.862	0.061	0.047	18.6	19.070
	3588	0.015	0.666	0.319	2.087	0.858	0.059	0.045	22.5	20.978
	4292	0.013	0.672	0.315	2.134	0.873	0.056	0.043	22.8	23.547
2	0	0.562	0	0.438	0	0	1	1	0	0.00
	6	0.325	0.268	0.407	0.659	0.555	0.783	0.679	0.8	0.279
	31	0.165	0.544	0.291	1.87	0.849	0.68	0.617	2.2	1.371
	101	0.111	0.602	0.286	2.103	0.869	0.488	0.424	4.7	3.914
	198	0.087	0.62	0.293	2.111	0.869	0.381	0.322	7.2	6.554
	363	0.063	0.639	0.298	2.146	0.868	0.271	0.221	9.6	9.638
	619	0.044	0.653	0.303	2.154	0.877	0.203	0.166	11.5	12.601
	981	0.033	0.661	0.306	2.163	0.871	0.145	0.115	13.7	15.066
	1386	0.03	0.664	0.306	2.172	0.871	0.133	0.105	17.7	16.760
	2015	0.019	0.673	0.307	2.189	0.88	0.091	0.073	18.5	18.422
	2970	0.017	0.677	0.306	2.216	0.883	0.08	0.064	21	20.011
	3588	0.014	0.681	0.305	2.234	0.883	0.067	0.053	21.3	20.772
	4292	0.011	0.685	0.304	2.253	0.886	0.055	0.044	20.5	21.510
3	0	0.538	0	0.462	0	0	1	1	0	0.00
	6	0.376	0.154	0.47	0.327	0.342	0.801	0.681	0.9	0.579
	31	0.209	0.466	0.325	1.433	0.775	0.709	0.633	2.7	2.621
	101	0.139	0.553	0.308	1.792	0.837	0.565	0.495	5.9	6.348
	198	0.106	0.58	0.314	1.85	0.84	0.434	0.366	8.8	9.204
	363	0.083	0.595	0.322	1.85	0.838	0.333	0.273	12.6	11.756
	619	0.057	0.615	0.328	1.875	0.852	0.247	0.202	14.3	13.755
	981	0.036	0.632	0.332	1.901	0.848	0.152	0.12	14.8	15.296
	1386	0.039	0.629	0.332	1.892	0.848	0.165	0.131	16.1	16.428
	2015	0.021	0.643	0.335	1.918	0.863	0.099	0.079	17.9	17.777
	2970	0.015	0.649	0.335	1.936	0.869	0.074	0.059	19	19.583
	3588	0.014	0.284	0.702	0.405	0.456	0.078	0.065	21.5	20.764
	4292	0.012	0.652	0.335	1.945	0.867	0.058	0.046	22	22.196

Unit of PAR: µmol m^−2^ s^−1^. Unit of ETR: μmol m^−2^ s^−1^.

The slow kinetics of chlorophyll fluorescence of *E. brevicornu* leaves indicates that the maximal photochemical efficiency of photosystem II ((Fm − Fo)/Fm) and the ETR of photosystem II in them were all very low.

The fraction of energy dissipated as heat via the regulated photoprotective NPQ mechanism (Y(NPQ)) was much more than that passively dissipated in the form of heat and fluorescence (Y(NO)).

**Figure 4:**
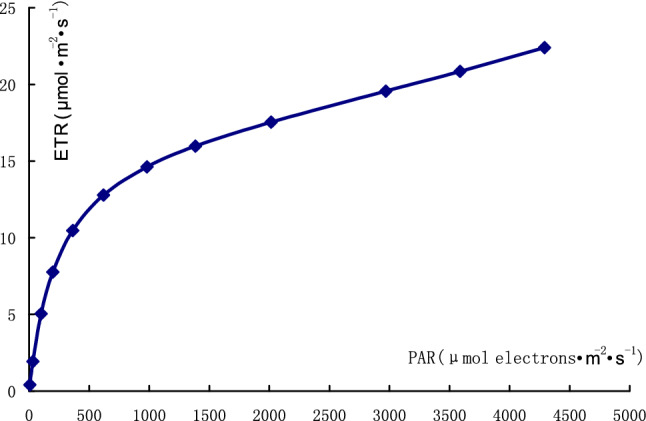
Fitted rapid light curve of chlorophyll fluorescence in *Epimedium pubescens* leaf with average indexes.

The results of rapid light curves of chlorophyll fluorescence in *E. brevicornu* leaves are shown in Tables 5 and 6. The fitted rapid light curve of chlorophyll fluorescence with the average fitted indexes in each repeat of rapid light curve is shown in Fig. 4.

**Table 6:** Results of fitting rapid light curves of chlorophyll fluorescence in *Epimedium pubescens* leaves.

Index	Repeat			Average
	1	2	3	
fv/fm × ETR factor/2	0.241	0.236	0.226	0.2343
Alpha	0.043	0.046	0.06	0.0497
ETRmax	21.4	19.4	25.8	22.2
Ik	493.1	425.6	427.2	448.63
(Fm − Fo)/Fm	0.5727	0.5616	0.5383	0.5575
A	−0.000006637	−0.0000009711	−0.000003762	−0.00000379
B	0.06827	0.04571	0.05887	0.0576
C	11.5297	21.197	10.0040	14.24
R^2^	0.993	0.987	0.952	0.977

fv/fm × ETR factor/2 is the maximum quantum yield of photosynthetic system II with a saturated pulse after dark adaptation. Alpha is the initial slope. ETRmax is the maximum electron transport rate. Ik is the minimum saturation of the light intensity.

The fitted maximum electron transport rate in the leaves reached 22.2 μmol m^−2^ s^−1^, which was much higher than ETR of photosystem II in slow kinetics of chlorophyll fluorescence. The leaves possessed large potential of photosynthesis.

## Discussion and conclusion

There was obvious midday depression in the photosynthesis of *E. brevicornu* leaves*.* The photosynthesis nearly stopped at noon when the direct sunlight was intense. Therefore, *E. brevicornu* could not endure strong sunlight and high air temperature. There was obvious midday depression in the photosynthesis of *E. pseudowushannense* also^5,6^. The midday depression in the photosynthesis of *E. brevicornu* leaves was related to sunlight and air temperature^7^. The net photosynthetic rate of *E. brevicornu* leaves significantly reduced when the sunlight intensity was above 1000 µmol m^−2^ s^−1^. There was little variation in sunlight intensity from 11:00 to 14:00 in summer. The net photosynthetic rates of *E. brevicornu* leaves was the lowest at 13:00 in summer because the air temperature was the highest at this time. The light compensation point of the leaves was about 15 µmol m^−2^ s^−1^, which indicated that *E. brevicornu* could survive in very low sunlight. The study of Liu et al. showed that the light compensation point of *E. sagittatum* (Sieb. & Zucc.) Maxim leaves was 13–17 µmol m^−2^ s^−1^^8^, which was consistent with the result in this study. The result of WANG et al. that the light compensation point of *E. sagittatum* was about 3.6 µmol m^−2^ s^−1^ seemed not reasonable^9^. The light saturation point in the light response curve of *E. brevicornu* leaves was lower than general sunlight intensity at noon in summer. Wild *E. brevicornu* grew in forests or shady slope and was rarely seen in the open places in general. Therefore, *E. brevicornu* is a typical shade plant. Luo et al. studied the characteristics of photosynthesis in *E. koreanum* Nakai and found that the photosynthetic rate was the highest in 70% light transmittance^10^. *E. brevicornu* should be properly shaded when cultivated.

It was indicated in CO_2_ response curve in *E. brevicornu* leaves that the CO_2_ saturation point was about 1766 µmol mol^−1^, which was much higher than the content of CO_2_ in general air. Therefore, *E. brevicornu* is characteristic of C_3_ plant. The net photosynthetic rate of *E. brevicornu* leaves was linearly correlated with the content of CO_2_ in air when the leaf chamber when it was below CO_2_ saturation point. This is consistent with the study of Wang Xujun on *E.* sagittatum^9^. The results indicated that there was very great potential to utilize CO_2_ in *E. brevicornu*.

The characteristics of chlorophyll fluorescence in *E. brevicornu* showed that it possessed great potential of photosynthesis. It is prospective to breed a new breed with high photosynthetic rate and yield.

## Materials and methods

All methods were performed in accordance with the local relevant guidelines, regulations and legislation.

### Instruments

LI-6400 photosynthesis system (LI-6400 Inc., Lincoln, NE, USA) and PAM-2500 portable chlorophyll fluorescence apparatus (PAM-2500, Walz, Germany) were used in the study.

### Materials

About 90 living *E. brevicornu* plants were collected from Taihang Mountains in October 2018. The *E. brevicornu* was not in endangered or protected. The collection of these *E. brevicornu* plants was permitted by local government. These plants were averagely planted in nine plots of 2 m^2^. The roots of *E*. *pubescens* were planted 6–8 cm below ground. These plots were placed on farmland near Taihang Mountains and covered with sunshade net (about 70% light transmittance). These plants were timely irrigated after planting to ensure that they grew well but not fertilized.

### Determination of photosynthetic characteristics

The photosynthetic characteristics of mature leaves on the *E. brevicornu* plants were determined between June 6–8, 2019 with the Li-6400 photosynthesis system. The diurnal variation of photosynthesis in three leaves of three plants was determined. When the light response curve was determined, the temperature of the leaf chamber was set at 28 °C, and the concentration of CO_2_ in the leaf chamber was set at 400 µmol mol^−1^. When determining the CO_2_ response curve, the light intensity in the leaf chamber was set at 1000 µmol m^−2^ s^−1^, and the temperature of the leaf chamber was set at 28 °C. The light response curve and CO_2_ response curve were determined three times in three leaves of three different plants.

### Determination of chlorophyll fluorescence characteristics

The fluorescence characteristics of chlorophyll in *E. brevicornu* leaves were determined with PAM-2500 portable chlorophyll fluorescence apparatus between June 8–9, 2019. The leaves underwent dark adaptation for 30 min before determining slow kinetics of chlorophyll fluorescence. Then the light curves of chlorophyll fluorescence were determined. All of these determinations were repeated three times on three mature leaves of three plants.

The data was analysed with SPSS (Statistical Product and Service Solutions, International Business Machines Corporation, USA). The light response curves were fitted with following modified rectangular hyperbola model^11,12^.

$${\text{Photo}}\, = \,{\text{E}}\cdot\left( {{1} - {\text{M}}\cdot{\text{PAR}}} \right)\cdot\left( {{\text{PAR}} - {\text{LCP}}} \right)/({1}\, + \,{\text{N}}\cdot{\text{PAR}})$$

PAR is the value of light intensity in leaf chamber of Li-6400 photosynthesis system. Photo is net photosynthetic rate. LCP is the light compensation point. E is the apparent quantum yield. M and N are parameters. The dark respiration rate under the LCP is calculated according to E·LCP. The light saturation point (LSP) is calculated according to (((M + N) ·(1 + N·LCP)/M)^½^)/−1)/N.

The net photosynthetic rate under the light saturation point (LSP) can be calculated according to the above model.

The CO_2_ response curves were fitted with below modified rectangular hyperbola model^11,12^.

$${\text{Photo}}\, = \,{\text{E}}\cdot\left( {{1} - {\text{M}}\cdot{\text{PAR}}} \right)\cdot\left( {{\text{PAR}} - {\text{CCP}}} \right)/({1}\, + \,{\text{N}}\cdot{\text{PAR}})$$

PAR is the value of light intensity in leaf chamber of Li-6400 photosynthesis system. Photo is net photosynthetic rate. CCP is CO_2_ compensation point. E is also the apparent quantum yield. M and N are parameters. The dark respiration rate under the CO_2_ calculated according to E·CCP. The CO_2_ saturation point (CSP) is calculated according to (((M + N) ·(1 + N·CCP)/M)^½^)/−1)/N.

The net photosynthetic rate under the CO_2_ saturation point (CSP) can be alternatively calculated according to the above model.

The light curves of chlorophyll fluorescence were fitted according to the below model of Eilers and Peeters^12,13^^.^

$${\text{ETR}}\, = \,{\text{PAR}}/({\text{a}}\cdot{\text{PAR}}^{{2}} \, + \,{\text{b}}\cdot{\text{PAR}}\, + \,{\text{c}})$$

ETR is the electron transport rate of photosynthetic system II. PAR is fluorescence intensity. The letters a, b and c are parameters.

## Data Availability

Data have been permanently archived: https://zenodo.org/record/4106097#.X41VfNSF7Gg.
